# miR-576-5p Promotes the Proliferation of Papillary Thyroid Carcinoma through the MAPK4-AKT Pathway

**DOI:** 10.1155/2022/1428411

**Published:** 2022-12-29

**Authors:** Rui Hai, Yang Zhou, Fan Li, Qingxi Guo, Yang Long, Fang Wang, Lian Cheng, Fei Wu, Shi Chen, Xiangyu Zhou

**Affiliations:** ^1^Department of Vascular, Breast, Thyroid Surgery, The Affiliated Hospital of Traditional Chinese Medicine of Southwest Medical University, Luzhou 646000, China; ^2^Vascular Surgery, Deyang People's Hospital, Deyang 618000, China; ^3^Department of Breast and Thyroid Surgery, Sichuan University Huaxi Guang'an Hospital, Guan'an 638500, China; ^4^Department of Pathology, The Affiliated Hospital of Southwest Medical University, Luzhou 646000, China; ^5^Experimental Medicine Center, The Affiliated Hospital of Southwest Medical University, Luzhou 646000, China; ^6^Department of General Surgery (Thyroid Surgery), The Affiliated Hospital of Southwest Medical University, Luzhou 646000, China

## Abstract

**Background:**

MicroRNA-576-5p (miR-576-5p) plays an important role in different human cancers. However, the biological function of miR-576-5p in papillary thyroid carcinoma (PTC) is still unclear. In this study, we explored the function and specific role of miR-576-5p in PTC.

**Methods:**

Expression levels of miR-576-5p in PTC patient tissues and cell lines were determined by reverse transcription-quantitative polymerase chain reaction (qRT‒PCR). Cell counting using cell counting kit-8 (CCK-8), wound healing, and Transwell assays were performed to evaluate the effect of miR-576-5p on the proliferation, migration, and invasion of TPC-1 cells. Expression levels of mitogen-activated protein kinase 4 (MAPK4) and phosphorylation levels of protein kinase B (AKT), extracellular regulated protein kinase (ERK), and P38 mitogen-activated protein kinase (P38) were detected by western blotting or immunohistochemistry (IHC).

**Results:**

The expression level of miR-576-5p in PTC tissues and TPC-1 cells was significantly increased. In vitro, overexpression of miR-576-5p promoted the proliferation, migration, and invasion of TPC-1 cells. In addition, MAPK4 was highly expressed in PTC tissues, and miR-576-5p could upregulate the expression of MAPK4. Interestingly, MAPK4 knockdown reversed cell proliferation but not migration and invasion in TPC-1 cells after miR-576-5p was overexpressed. Moreover, overexpression of miR-576-5p induced activation of the AKT pathway in TPC-1 cells, and MAPK4 gene knockout reversed this AKT pathway activation.

**Conclusion:**

In this study, we found that miR-576-5p was significantly overexpressed in PTC tissues and TPC-1 cells. In addition, miR-576-5p promoted the proliferation of TPC-1 cells by enhancing expression of MAPK4 and activating the AKT pathway.

## 1. Introduction

Thyroid cancer (TC) is the most common malignancy of the endocrine system, and its incidence has increased steadily over the past decade worldwide [1]. According to different pathological features, thyroid cancer can be divided into papillary thyroid carcinoma (PTC), follicular thyroid carcinoma (FTC), medullary thyroid carcinoma (MTC), and anaplastic thyroid cancer (ATC) [2, 3]. PTC is the most common pathological type of TC, accounting for more than 80% of TC cases. PTC is generally considered to be a type of malignancy with a slow progression and good prognosis, as the 10-year survival rate is approximately 90% [4]. However, in some patients, PTC shows aggressive behaviour, resulting in poor prognosis. Although the overall survival rate improves significantly after surgery or radiotherapy, the prognosis of metastatic PTC is poor [5]. Thus, accurate preoperative diagnosis and precise treatment for PTC are necessary. The discovery of specific biomarkers in PTC can help to evaluate the invasive potential of tumours and guide surgical strategies or provide new therapeutic targets [6]. Therefore, it is of great significance to elucidate the molecular mechanism by which PTC develops to obtain new therapeutic targets.

Mutations or abnormalities of microRNAs (miRNAs), which are a group of small (18–25 nucleotides in length) highly conserved noncoding RNA molecules that play a key role in tumorigenesis, development, invasion, and metastasis, may lead to cancer [7, 8]. A large number of miRNAs, such as miR-622, miR-214, and microRNA-23a, have been found to be involved in regulating the occurrence and development of papillary thyroid carcinoma [9–11]. In addition, epigenetic alteration of cell function in PTC has yet to be fully understood. miR-576-5p has been found to be upregulated in various cancers, such as glioblastoma and colon cancer [12, 13], and overexpression of miR-576-5p has been associated with brain metastasis of colorectal cancer and survival in pulmonary neuroendocrine tumour [14, 15]. Furthermore, it has been proven that miR-576-5p can promote proliferation, migration, and invasion in nonsmall cell lung cancer cells [16]. However, to the best of our knowledge, the specific roles and mechanisms of miR-576-5p in PTC have not yet been explored, and in light of this, we designed this study and performed a series of experiments to analyse the biological roles of miR-576-5p and associated mechanisms in TPC-1 cells.

As an atypical member of the MAPK family, the physiological and pathological functions of mitogen-activated protein kinase 4 (MAPK4) in tumours have gradually begun to gain attention. MAPK4 has been shown to promote breast cancer cell proliferation, migration, and invasion by activating PI3K/AKT signalling, the downstream protein c-JUN, the G1/S cell cycle, and the epithelial-to-mesenchymal transition (EMT). At the same time, MAPK4 is highly expressed in osteosarcoma and inhibits cell proliferation and migration by activating the JNK/p38 signalling pathway. In prostate cancer studies, MAPK4 overexpression promoted prostate cancer metastasis through HSP27 upregulation. In addition, MAP2K4 can activate the p38 protein and induce prostate cancer epithelial cells to transform into mesenchymal cells, thus leading to distant tumour cell metastasis [17, 18]. However, MAPK4 was found to be downregulated in pancreatic adenocarcinoma and identified as a tumour suppressor. [19] Despite the differences in expression in the above studies, the role of MAPK4 in PTC and its relationship with the AKT signalling pathway are still unclear.

In this study, we evaluated the effect of miR-576-5p on the proliferation, migration, and invasion of papillary thyroid carcinoma. Additionally, we investigated whether the MAPK4-AKT signalling pathway mediates the role of miR-576-5p in promoting tumour proliferation, migration, and invasion.

## 2. Materials and Methods

### 2.1. PTC Tissue Sample Collection

PTC tissues and adjacent normal thyroid tissues were collected before surgery from patients diagnosed with PTC who then underwent thyroidectomy from July 2018 to April 2019 at the Affiliated Hospital of Southwest Medical University. To ensure that there were sufficient samples for the final pathological diagnosis, 42 pairs of PTC tissues and normal tissue samples were collected immediately after thyroidectomy, approximately 2 g each. The samples were immediately frozen in a liquid nitrogen at −180°C and then transferred to a cryogenic freezer at −80°C for long-term storage. All samples were collected with informed consent from the patients and their families, and relevant ethics documents were signed. The experiment was approved by the ethics committee of the Affiliated Hospital of Southwest Medical University.

### 2.2. Cell Culture

Human thyroid papillary carcinoma cells (TPC-1) and normal thyroid epithelial cells (Nthy-ori 3-1) were obtained from Guangzhou Gino Biotechnology Co., Ltd. of China. TPC-1 cells were cultured in RPMI 1640 medium (HyClone Company, USA) supplemented with 10% foetal bovine serum (FBS, Gibco, Thermo Fisher Company, USA) and incubated at 37°C in a humidified incubator with 5% CO_2_ (Thermo Fisher, USA). Nthy-ori 3-1 cells were cultured in F-12K medium (Gibco, Thermo Fisher Company, USA) supplemented with 10% FBS (Gibco, Thermo Fisher Company, USA) at 37°C and 5% CO_2_.

### 2.3. Cell Transfection

miR-576-5p mimic (5′-AUUCUAAUUUCUCCACGUCUUU-3′; 50 nmol/L), miR-576-5p inhibitor (5′-AAAGACGUGGAGAAAUUAGAAU-3′; 100 nmol/L), mimic negative control (NC; 5′-UUUGUACUACACAAAAGUACUG-3′; 50 nmol/L), inhibitor negative control (NC; 5′-CAGUACUUUUGUGUAGUACAAA-3′; 100 nmol/L), and siRNA-MAPK4(GGCGCTTTGTTGACTTCCA; 100 nmol/L) were used in this study. All oligonucleotides were purchased from Ribose Biology Co., Ltd. (Guangzhou, China). Lipofectamine 2000 (Invitrogen, USA), which is a multifunctional transfection reagent that can effectively transfect various cargo into various adherent and suspension cell lines, was used as the transfection reagent. According to the instructions, cells were inoculated into 12-well plates one day before transfection, and transfection was carried out when the cell density reached 50%. The final concentration of the miR-576-5p mimic and mimic negative control was 50 nmol; that of the miR-576-5p inhibitor and inhibitor negative control was 100 nmol. Transfected cells were cultured at 37°C in a humidified incubator containing 5% CO_2_, and the medium was changed after 24 hours. Then, the cells were used for experiments.

### 2.4. RNA Extraction and Reverse Transcription-Quantitative Polymerase Chain Reaction (RT-qPCR) Analysis

Total RNA of patient tissues and cell lines was extracted with TRIzol reagent (Thermo Fisher, USA); the nucleic acid quantity, quality, and purity were determined using a spectrophotometer (Nanodrop, Thermo Fisher Company, USA) and 1% agarose gel electrophoresis. Subsequently, 500 ng of RNA was used for reverse transcription (Catalogue No. 218160; Chagan Co., Ltd., Hilden City, Germany). The cDNA obtained was used for RT-qPCR using a SYBR green PCR kit according to the manufacturer's instructions (product catalogue No. 208054; German Hilden Chagan Co., Ltd.). Raw materials for miR-576-5p and U6 were purchased from Chagan Company (product catalogue No. MIMAT0018987; MS00044996). RT-qPCR was performed using a StepOnePlus version 2.2.3 A real-time PCR system (Applied Biosystems; Thermo Fisher Scientific, Inc.). Expression of miR-576-5p was calculated relative to that of U6 using the comparative threshold method (2^−∆∆ct^).

### 2.5. Cell Wound Healing

Cell wound healing was used to evaluate the migration of TPC-1 cells. Briefly, transfected TPC-1 cells (5 × 10^5^ cells/well) were inoculated into 24-well plates and cultured in serum-free RPMI 1640 medium; the cell inoculation density was approximately 30%. When the cells completely covered the bottom of the well, a scratch was made perpendicular to the bottom of the well to form a linear wound using a pipette tip. Then, the scratched cells were washed with PBS and cultured in serum-free RPMI 1640 medium for 24 hours. The wound closure distance was measured by photographing five randomly selected areas at the time of injury (time 0) and at 24 hours after injury.

### 2.6. Transwell Assay

Transwell assays were used to detect the invasiveness of cells. Briefly, we seeded cells in 24-well plates without serum medium but containing Matrigel-coated insert filters (Costar Corning, USA), and then RPMI 1640 medium with 10% FBS was added to the lower chamber. The cells were cultured at 37°C in 5% CO_2_ for 24 hours, fixed with 4% formaldehyde through a filter for 10 minutes, and stained with 5% crystal violet. We quantitatively analysed the cells invading the Matrigel membrane.

### 2.7. Cell Counting Kit-8 (CCK8) Proliferation Assays

Cell proliferation was assessed by the CCK8 assay (Japan Co.). TPC-1 cells (3 × 10 [3] cells/well) were inoculated into 96-well plates in a final volume of 100 *μ*l and then transfected with the abovementioned miR-576-5p mimic, inhibitor, and corresponding NC. Samples were assessed at 24, 48, and 72 hours after transfection. CCK-8 solution (10 *μ*l) was added to each well and incubated at 37°C for 2 hours. Absorption was measured at 450 nm to calculate the number of viable cells.

### 2.8. Immunohistochemistry (IHC) and Western Blot Analysis

Analysis of MAPK4 expression in formalin-fixedparaffin-embedded tissue 4 *μ*m sections was performed by IHC, and expression of MAPK4 was examined by using an anti-MAPK4 antibody (1 : 50, ab2011501; Abcam company, Cambridge, Massachusetts). For western blotting, total protein was extracted from TPC-1 cells or PTC tissues using RIPA buffer (Beyotime Institute of Biotechnology, China). The total protein lysates were separated by 10% sodium dodecyl sulfate-polyacrylamide gel electrophoresis and transferred to a polyvinylidene fluoride (PVDF) membrane. After that, the membranes were blocked at room temperature for 2 hours using skim milk and then incubated with primary antibodies (anti-MAPK4_1 : 1000; anti-PCNA_1 : 1000; anti-p-AKT_1 : 1000; anti-AKT_1 : 1000; anti-p-ERK_1 : 1000; anti-ERK_1 : 1000; anti-p-P3_1 : 1000; anti-P38_1 : 1000; anti-GAPDH_1 : 2000) overnight at 4°C. The next day, the PVDF membrane was washed three times with PBST and then incubated at room temperature for 1 hour with 1 : 3000 diluted antirabbit IgG (Beyotime Institute of Biotechnology, China; cat: A0208) and 1 : 3000 diluted antimouse IgG (Beyotime Institute of Biotechnology, China; cat: A0216). Protein bands were detected using Enhanced Chemiluminescence Detection Reagent (Bio-Rad Laboratories, Inc., Hercules, CA, USA.) and quantitatively analysed by Photoshop image software.

### 2.9. Animal Studies

Twelve male BALB/c nude mice (3-4 weeks old) with similar body weights were obtained from Chongqing Tengxin company and randomly divided into 4 groups, 3 in each group. A total of 5 × 10^6^ transfected TPC-1 cells (miR-576-5p mimic, miR-576-5p mimic NC, miR-576-5p inhibitor, and miR-576-5p inhibitor NC) were injected subcutaneously into the armpits twice a week for 4 weeks until euthanasia under anaesthesia. Tumour size was measured with a calliper, and the tumour volume (V) was calculated according to the formula *V*=(*L* x *W*^2^)/2. After 4 weeks, the tumour was removed, weighed, and quickly stored at −80°C for further analysis [20]. According to the programme approved by the ethics committee of Southwest Medical University, animal experiments and use are in accordance with the guidelines for animal experiments and use.

### 2.10. Statistical Analysis

All values are presented as the mean ± standard deviation (*n* = 3) and were processed by GraphPad Prism 6 (GraphPad, CA, USA). Differences between more than two groups were analysed by one-way ANOVA. Student's *t*-test was used to evaluate differences between the two groups. *P* < 0.05 was considered to be a significant difference.

## 3. Results

### 3.1. miR-576-5p Is Upregulated in PTC Tissues and TPC-1 Cells

**Figure 1:**
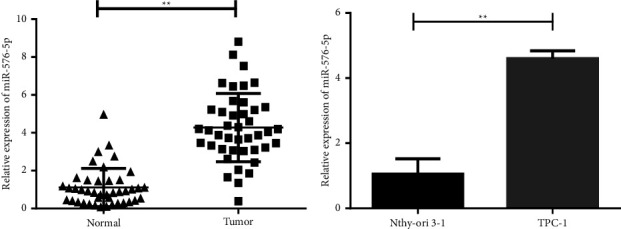
miR-576-5p was significantly upregulated in PTC tissues and TPC-1 cells. (a) Relative expression levels of miR-576-5p in PTC tissues and corresponding adjacent normal tissues were detected by RT-qPCR. (b) Relative expression levels of miR-576-5p in the PTC cell line TPC-1 and in the normal thyroid follicular epithelial cell line Nthy-ori 3-1 were detected by RT-qPCR. ^*∗∗*^*P* < 0.01.

To investigate miR-576-5p in tissues and cell lines, its expression in 42 pairs of human PTC tissues and adjacent normal tissues was detected by RT-qPCR. The results demonstrated that miR-576-5p expression was significantly upregulated in PTC tissues compared with adjacent normal tissues (Figure 1(a)). The median expression level of miR-576-5p (4.28) was taken as the cut-off value, and 42 PTC patients were divided into a low expression group (*n* = 24) and a high expression group (*n* = 18) according to the cut-off value of miR-576-5p. It should be noted that the expression level of miR-576-5p correlated with age (*P* < 0.05), TNM stage (*P* < 0.01), and lymph node metastasis (*P* < 0.01) (Table 1). Moreover, miR-576-5p was significantly overexpressed in TPC-1 cells compared with Nthy-ori 3-1 cells (Figure 1(b)). These data indicate that expression of miR-576-5p is increased in tissues and TPC-1 cells, suggesting that miR-576-5p may play an important role in the occurrence and development of PTC.

### 3.2. miR-576-5p Promotes the Migration, Proliferation, and Invasion of TPC-1 Cells

**Figure 2:**
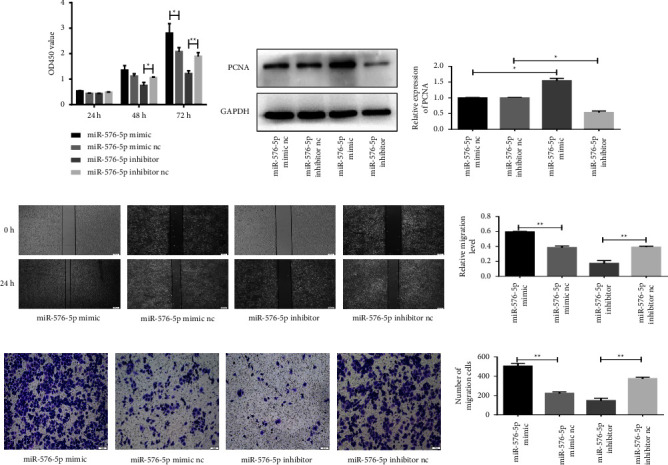
Overexpression of miR-576-5p promotes the proliferation, migration, and invasion of TPC-1 cells. (a) A CCK-8 assay was performed to evaluate cell proliferation. (b, c) The proliferation-related protein PCNA was evaluated by western blotting. (d, e) The effect of miR-576-5p on the migration of TPC-1 cells was examined by wound healing assays. (f, g) The effect of miR-576-5p on the invasion of TPC-1 cells was examined by transwell assays. ^*∗*^*P* < 0.05;  ^*∗∗*^*P* < 0.01.

Next, to investigate the role of miR-576-5p in PTC, a series of functional experiments were carried out by transferring miR-576-5p mimic, inhibitor, and corresponding negative controls into TPC-1 cells. The results from the CCK8 assay showed that upregulation of miR-576-5p promoted the proliferation of TPC-1 cells but downregulation of miR-576-5p inhibited cell proliferation (Figure 2(a)). At the same time, the expression level of the proliferation-related protein PCNA was increased after transfecting the miR-576-5p mimic and significantly decreased in TPC-1 cells with inhibited expression of miR-576-5p (Figures 2(b) and 2(c)). In addition, the results from cell wound healing and Transwell assays all showed that upregulation of miR-576-5p promoted the migration and invasion of TPC-1 cells and miR-576-5p downregulation inhibited these processes, as shown in Figures 2(d)–2(g). These insights show that miR-576-5p may play an important role in the proliferation, migration, and invasion of TPC-1 cells.

### 3.3. miR-576-5p-Induced Proliferation, Migration, and Invasion in TPC-1 Cells Is Dependent on AKT Signalling Pathway Activation

**Figure 3:**
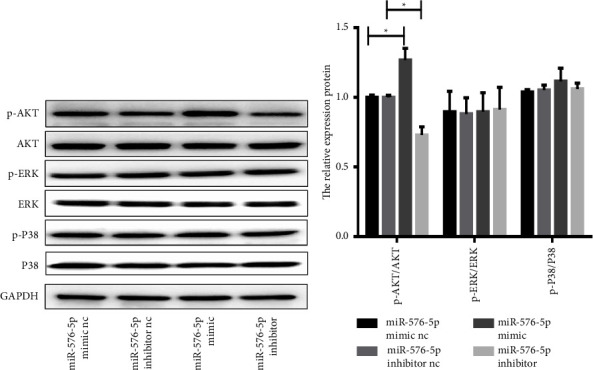
miR-576-5p correlated positively with phosphorylated AKT. Phosphorylation of AKT, ERK, and P38 was examined in TPC-1 cells by western blotting. ^*∗*^*P* < 0.05.

It has been demonstrated that both the MAPK and phosphatidylinositol 3-kinase (PI3K)/AKT signalling pathways regulate the oncogenic transformation, growth, and survival of cancer cells [21]. The key proteins of the two signalling pathways, including AKT, ERK, and P38, were detected to evaluate the effects of miR-576-5p on TPC-1 cell behaviours. Compared with the control group of miR-576-5p mimic, the level of phosphorylated AKT was significantly increased in miR-576-5p mimic-transfected TPC-1 cells, and the opposite effects were induced by its inhibitor. No obvious expression changes in ERK or P38 were found (Figure 3). These results suggest that miR-576-5p regulates the function of TPC-1 cells through the AKT signalling pathway, at least in part.

### 3.4. MAPK4 Downregulation Mediates miR-576-5p-Induced AKT Activation and Cell Proliferation in TPC-1 Cells

**Figure 4:**
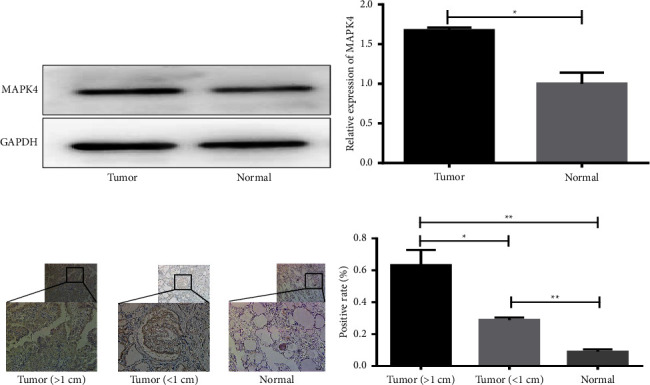
The expression level of MAPK4 in PTC was significantly higher than that in normal thyroid tissue. (a–d) Expression of MAPK4 in PTC and normal thyroid tissue by western blotting and immunohistochemistry. ^*∗*^*P* < 0.05;  ^*∗∗*^*P* < 0.01.

Emerging evidence indicates that MAPK4 regulation of the AKT signalling pathway may be involved in the pathogenesis of tumours, such as lung adenocarcinoma, colon cancer, and prostate cancer [18]. Based on these findings, we preliminarily hypothesized that miR-576-5p-induced functional changes in TPC-1 cells may occur through the MAPK4-AKT signalling axis. To test this hypothesis, expression of MAPK4 was first observed in clinical PTC specimens, and western blot results showed that the level of MAPK4 expression in PTC tissues was significantly higher than that in adjacent normal thyroid tissues, and similar data were found by IHC (Figures 4(a) and 4(b)). Moreover, IHC showed MAPK4 to be mainly expressed in the cytoplasm of thyroid cells. Interestingly, there were differences in tumour size, with rates of MAPK4 positivity in PTC (>1 cm), PTC (<1 cm), and normal thyroid tissues of 63.31%, 25.78%, and 9%, respectively (Figures 4(c) and 4(d)). These results suggest that MAPK4 is overexpressed and may play an important role in the development of PTC and may be related to the size of tumours.

**Figure 5:**
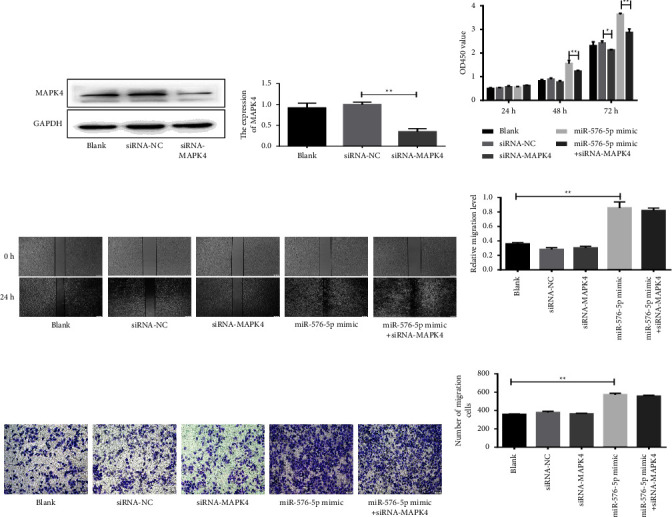
MAPK4 knockout inhibited the proliferation of TPC-1 cells. (a, b) MAPK4 expression in TPC-1 cells transfected with siRNA-MAPK4 and siRNA-NC. (c–g) The effect of MAPK4 on the proliferation, migration, and invasion of TPC-1 cells was examined by CCK8, wound healing, and transwell assays. ^*∗*^*P* < 0.05; ^*∗∗*^*P* < 0.01.

Next, we verified whether MAPK4 is involved in miR-576-5p-induced functional changes in vitro. As shown in Figures 5(a) and 5(b), expression of MAPK4 was significantly reduced in cells transfected with siRNA-MAPK4. CCK8 results showed that the proliferation ability of siRNA-MAPK4-transfected TPC-1 cells was significantly lower than that of siRNA-NC-transfected cells. Moreover, upregulation of miR-576-5p obviously reversed the effect of silencing MAPK4 on the proliferation of TPC-1 cells (Figure 5(c)). Unexpectedly, there was no effect on the migration or invasion of TPC-1 cells after silencing MAPK4, suggesting that MAPK4 may not participate in this process (Figures 5(d)–5(g)). These results indicate that MAPK4 is involved in modulating the proliferation but not the migration and invasion of TPC-1 cells and that this process is regulated by miR-576-5p.

**Figure 6:**
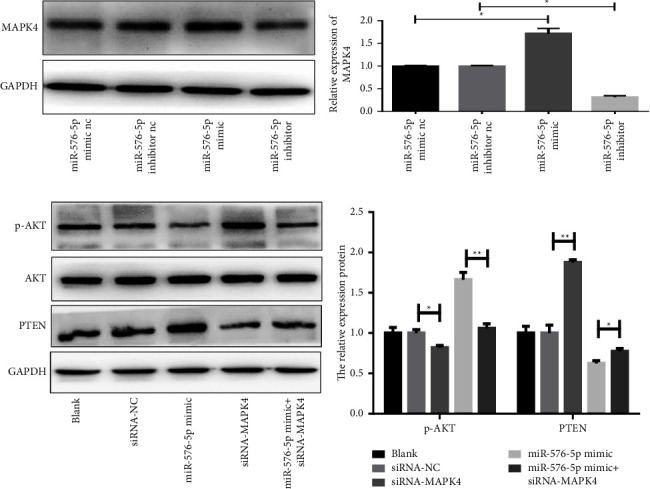
miR-576-5p promoted activation of MAPK4-AKT pathways in TPC-1 cells. (a–d) Protein expression levels of MAPK4 and PTEN/p-AKT were detected by western blotting. ^*∗*^*P* < 0.05; ^*∗∗*^*P* < 0.01.

Additionally, the regulatory relationship between MAPK4 and miR-576-5p in TPC-1 cells was investigated. MAPK4 expression was significantly upregulated after miR-576-5p mimic transfection and downregulated after miR-576-5p inhibitor transfection (Figures 6(a) and 6(b)). As expected, silenced expression of MAPK4 resulted in upregulation of PTEN protein levels and downregulation of p-AKT protein levels. At the same time, upregulation of miR-576-5p reversed the effect of MAPK on PTEN and p-ATK (Figures 6(c) and 6(d)).

### 3.5. miR-576-5p Promotes Tumour Growth In Vivo

**Figure 7:**
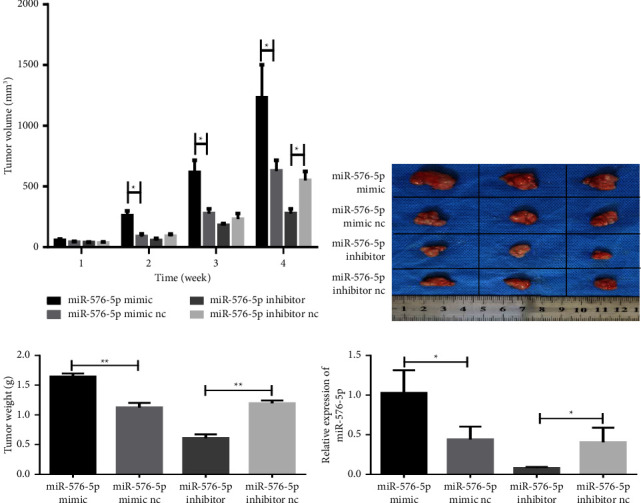
miR-576-5p promotes tumour growth in vivo. (a) Tumour volume was measured weekly after injection of transfected TPC-1 cells. (b) After the mice were killed, the tumour tissue was imaged. (c) The tumour weight was measured after the mice were killed. (d) Expression of miR-576-5p in tumour tissues was detected by RT‒qPCR. ^*∗*^*P* < 0.05;  ^*∗∗*^*P* < 0.01.

To evaluate whether miR-576-5p promotes tumour growth in vivo, we conducted animal experiments. The results showed that after upregulation of miR-576-5p, the growth rate of tumours was significantly increased. At the same time, after downregulation of miR-576-5p, the tumour growth rate was significantly decreased (Figure 7(a)). Four weeks later, the mice were killed, and the tumours were removed and weighed. The results showed that after upregulating miR-576-5p, the tumour size and weight were significantly increased. At the same time, after downregulating miR-576-5p, the tumour size and weight decreased significantly (Figures 7(b) and 7(c)). In addition, we detected expression of miR-576-5p in the tumour tissues, and the results showed that expression of miR-576-5p in the miR-576-5p mimic group was significantly higher than that in the miR-576-5p mimic NC group, but miR-576-5p expression in the miR-576-5p inhibitor group was significantly lower than that in the miR-576-5p inhibitor NC group (Figure 7(d)). These results indicate that miR-576-5p can promote tumour growth in vivo.

## 4. Discussion

Recently, a large number of miRNAs have been reported to be involved in the genesis, development, and prognosis of PTC [22–24]. In this study, we observed high expression of miR-576-5p in PTC tissues and TPC-1 cells. We regulated expression of miR-576-5p in TPC-1 cells in vitro and in vivo and found that upregulation of miR-576-5p can promote the proliferation, migration, and invasion of TPC-1 cells as well as the growth of tumours in vivo. Additionally, miR-576-5p resulted in upregulated expression of MAPK4 and enhanced AKT activity in TPC-1 cells. Finally, we confirmed that miR-576-5p-induced cell proliferation is mediated by the MAPK4-AKT signalling pathway in TPC-1 cells.

miR-576-5p has been reported to be associated with certain chronic inflammatory diseases, such as osteoarthritis, pertussis, systemic lupus erythematosus, nonalcoholic fatty liver disease, and tuberculosis [25–29]. Furthermore, miR-576-5p is upregulated in patients with colorectal cancer with brain metastases, glioblastoma, neuroendocrine tumours of the lung, and colon cancer [12–15]. These studies suggest that miR-576-5p correlates with tumorigenesis, distant metastasis, and overall survival of cancers and plays a key role in the function of cancer cells. Upregulated miR-576-5p promotes the migration and invasion of oesophageal squamous cell carcinoma (ESCC) by inhibiting expression of nuclear receptor interacting protein (NRIP1) [30]. It has been confirmed that miR-576-5p accelerates the invasion of various human melanoma cell lines [31]. Additionally, miR-576-5p is involved in regulating the proliferation of oesophageal cancer cells [32]. Consistent with previous studies, we found miR-576-5p to be highly expressed in PTC tissues and TPC-1 cells, and overexpression of miR-576-5p promoted the proliferation, migration, and invasion of TPC-1 cells.

The MAPK and PTEN/AKT signalling pathways play central roles in promoting cell proliferation [21, 33]. Many studies have shown that miRNAs regulate the proliferation, migration, and invasion of PTC through the MAPK and PTEN/AKT signalling pathways. For example, miR-150-5p promotes PTC cell proliferation and survival by activating the ERK signalling pathway [34]. miR-20b plays an inhibitory role in papillary thyroid carcinoma by regulating the MAPK/ERK signalling pathway[35]. Overexpression of miR-31 can significantly inhibit the proliferation, invasion, and migration of PTC cells by regulating the extracellular AKT signalling pathway [36]. Additionally, miR-486 inhibits cell proliferation, invasion, and migration by downregulating TENM1 expression and affecting the ERK and AKT signalling pathways and epithelial-to-mesenchymal transition in papillary thyroid carcinoma [37]. We also verified whether MAPK and/or PTEN/AKT are involved in the proliferation, migration, and invasion of TPC-1 cells with the participation of miR-576-5p and found that among key MAPK and AKT pathway components, only AKT protein expression changed with miR-576-5p knockdown. These data clearly confirm that the AKT pathway, and not MAPK signalling, is involved in miR-576-5p-induced cell functional regulation in PTC.

MAPK4 was initially identified as a negative regulator of proliferation in noncancer cells such as preadipocytes and multiple myeloma cell lines [38]. Recently, research on MAPK4 in the field of tumours has received increasing attention. In osteosarcoma-derived U2OS and ovarian carcinoma-derived ES-2 cells, decreased MAPK4 mRNA translation mediates the prometastatic effect of IGF2BP1, suggesting that MAPK4 acts as a tumour suppressor [39]. Conversely, MAPK4 is regarded as a tumour promoter in some cancers, such as lung adenocarcinoma, colon cancer, and prostate cancer [18]. Furthermore, the transcript levels of MAPK4 are upregulated by the oncogenic K-ras gene in lung adenomas [40]. A proliferative effect of MAPK4 was also observed in prostate cancer PC3 cells [18]. It seems that MAPK4 may exert different effects on different tumours, with anti- or pro-oncogenic effects. In our study, we verified that MAPK4 was overexpressed in PTC tissues. Additionally, we found that MAPK4 could promote the proliferation of papillary thyroid cancer cells and mediate the effect of miR-576-5p on the proliferation of TPC-1 cells.

As mentioned above, miR-576-5p is definitely involved in the regulation of the function of PTC cells, as induced by MAPK4. To investigate a possible interaction between miR-576-5p and MAPK4, miR-576-5p gain- and loss-of-function experiments in TPC-1 cells were performed. Our data showed that overexpression of miR-576-5p led to upregulation of MAPK4 and that inhibiting miR-576-5p prevented it. These findings confirm that miR-576-5p directly or indirectly activates MAPK4 to promote cell proliferation in PTC, though the detailed regulatory mechanisms need to be further confirmed.

It was shown that the MAPK4 and AKT signalling pathways are related in a variety of tumours. Wang et al. suggested that MAPK4 promotes the progression of both lung cancer and bladder cancer through activation of the AKT signalling pathway [18]. Similar to the above studies, as expected, silenced expression of MAPK4 resulted in upregulation of PTEN protein levels and downregulation of p-AKT protein levels. At the same time, upregulation of miR-576-5p reversed the effect of MAPK on PTEN and p-ATK. Together, these data suggest that miR-576-5p regulates cells through the MAPK4-AKT pathway, but there is no evidence of direct regulation between miR-576-5p and MAPK4. Whether other mechanisms are involved in miR-576-5p-mediated functional changes in thyroid carcinoma cells remains to be further explored.

In summary, our study showed that expression of miR-576-5p was significantly increased in PTC tissues and TPC-1 cells. Furthermore, we found that miR-576-5p can promote the proliferation of TPC-1 cells by enhancing expression of MAPK4 and activating the downstream AKT signalling pathway. The direct target gene of miR-576-5p will be the focus of our future research.

## Figures and Tables

**Table 1:** Correlation between clinicopathological features and miR-576-5p expression in 42 patients with PTC.

Characteristics	*n*	High expression	Low expression	*P* value
Sex				0.367
Male	12	5	7	
Female	30	13	17	
Age, years				0.023
<55	28	10	18	
≥55	14	8	6	
Extrathyroidal extension				0.131
Yes	22	11	11	
No	20	7	13	
TNM staging				0.008
I-II	26	9	17	
III-IV	16	9	7	
Lymph node metastasis				0.007
Yes	24	13	11	
No	18	5	13	
Multifocality				0.166
Yes	19	7	12	
No	23	11	12	
Tumour size, cm				0.103
<2	31	12	19	
≥2	11	6	5	

## Data Availability

The figure data and related data used to support the findings of this study are included within the article.
